# Response to Comment on “The effect of disease-modifying anti-rheumatic drugs on skeletal muscle mass in rheumatoid arthritis patients: a systematic review with meta-analysis”

**DOI:** 10.1186/s13075-022-02932-5

**Published:** 2022-11-01

**Authors:** Thales R. Hein, Leonardo Peterson, Barbara J. Bartikoski, Juliana Portes, Rafaela C. Espirito Santo, Ricardo M. Xavier

**Affiliations:** Universidade Federal Do Rio Grande Do Sul, Rheumatology, Rua Ramiro Barcelos, 2350, Porto Alegre, RS 90035‑903 Brazil

Dear authors,

Thank you for reading our paper and for the critical analysis. All suggestions are relevant, and the issues were deeply discussed by our group to solve them.

About the first point raised by the authors, we agreed that we had lesser papers about anti-TNF therapy included. However, we revised our inclusion/exclusion criteria, and we decided to still not include the mentioned papers. These studies presented relevant data, but methodological issues such as therapy regimen force us to exclude them.

The second point, about why we did not perform our analysis with the valued adjusted by size, is relevant and must be discussed. The main problem is because not all studies collected or mentioned that data, so the number of studies in the meta-analysis would significantly decrease. Still, we agreed that adjusted muscle mass and not adjusted muscle mass are different measures, and we are planning to perform this analysis to add in a possible reply to this letter.

Finally, with the last point, we agreed that DEXA and BIA are different methods of body composition measure and that could lead to confusion in results. Our group decided to perform another meta-analysis separating studies that used DEXA from studies that used BIA and add that data in a reply to this letter.

